# Breast cancer and occupational exposures: an integrative review of the literature

**DOI:** 10.47626/1679-4435-2020-595

**Published:** 2021-03-03

**Authors:** Arthur Brito-Marcelino, Rodrigo Japur Duarte-Tavares, Katienne Brito Marcelino, Julio Alves Silva-Neto

**Affiliations:** 1 Fonoaudiologia, Hospital de Urgência de Sergipe, Aracaju, SE, Brazil; 2 Fundação Oswaldo Cruz (Fiocruz), Rio de janeiro, RJ, Brazil; 3 Departamento de Farmácia, Universidade de São Paulo, Ribeirão Preto, SP, Brazil; 4 Departamento de Medicina, Universidade de Gurupi, Gurupi, TO, Brazil

**Keywords:** cancer, breast cancer, workers’ health, occupational illness

## Abstract

Occupational factors can lead to breast cancer, though the relationship between these variables is not well established. The objective of this study was to search the relevant literature for information on the association between breast cancer and exposure to occupational risk factors. For that purpose, electronic databases were searched using the following keywords: breast cancer and occupational exposures. A total of 40 articles published in the 10-year period from 2009 to 2019 were included in this review. Workers exposed to metals such as cadmium, chemical products, radiation and night work were more susceptible to breast cancer. The findings showed significant evidence to support an association between breast cancer and some chemical products, ionizing radiation and night work. However, most studies have difficulty establishing a causal relationship between these variables, pointing to the need for further investigation of these issues.

## INTRODUCTION

Breast cancer accounts for 25% of malignancies in women.^1-3^ It is one of the two most common causes of cancer death in this population, second only to lung cancer.^1,4^ In Brazil, breast cancer is the most frequent malignancy, accounting for 22% of cancers in women.^1^ In 2019, nearly 57,900 new cases of breast cancer were diagnosed in the country.^2^ Though strategies for the early diagnosis and prevention of breast cancer have been implemented around the world, this condition is still associated with a high risk of death.^5^

The etiology of breast cancer is not fully understood, and many possible causal factors are ambiguous and poorly defined.^3,6^ Nevertheless, the pathogenesis of breast cancer is known to be multifactorial, with risk factors that include genetic predisposition, heredity, reproductive factors, alcohol intake, smoking, overweight and hormone therapy.^4,7-9^ Yet these factors alone cannot explain all cases.

There has been a significant increase in global cancer rates, especially in countries with rapidly developing human, economic and industrial activities.^5,8^ Studies have observed that the daughters of women who emigrate from countries with low rates of breast cancer show a high incidence of this condition, with rates that resemble those observed in the country of immigration.^8^ This finding demonstrates that lifestyle and environmental or occupational factors can also lead to an increase in breast cancer cases.^3^

The association between breast cancer and occupational factors is a long-studied issue in the literature. In the early 18th century, Bernardino Ramazzini found that the prevalence of breast cancer was higher among nuns than in the general population. Ramazzini attributed this association to the celibate lifestyle of the nuns.^10^

In recent years, technological developments have allowed for a more thorough study of the association between breast cancer and occupational factors.^3,8^ It is estimated that 8 to 16% of cancers are caused by occupational exposures.^2^ This phenomenon is caused by the presence of carcinogens in the work environment.^1^ Carcinogenic compounds include, but are not limited to, organic fibers and dust particles (asbestos, silica and wood dust), metals (lead, cadmium, beryllium, chrome, cobalt and nickel), solvents (benzene and trichloroethylene), mineral oils (e.g., petrochemical and combustion products, diesel exhaust), reactive chemical products (ethylene, formaldehyde, vinyl chloride, strong inorganic acid mists containing sulfuric acid, aromatic amines and insecticides).^1^

In the context of breast cancer, some studies have assessed occupational exposures that may increase the risk of this condition in specific occupations. Sources of exposure include ionizing and non-ionizing radiation, melatonin suppression in night workers, pesticides, solvents, aromatic hydrocarbons, some metals and other chemical substances.^3,5,6,8,11^

Despite the long history of research on breast cancer and the fact that some substances are proven carcinogens, the *International Agency for Research on Cancer* (IARC) has stated that there is still a need for further evidence of the association between occupational risk factors and breast cancer in humans.^5^

Therefore, the aim of this study was to conduct an integrative literature review on the association between breast cancer and occupational exposures.

## METHODS

An integrative literature review method was used. The study was conducted in the following stages: research question, literature search, data collection, data analysis, interpretation of results, discussion and presentation of the findings.

This review was guided by the following questions: is breast cancer related to occupational risk factors? And if so, which occupational risk factors contribute to the development of cancer?

The PubMed, SciELO and LILACS databases were searched using the following keywords: “breast cancer” and “occupational exposures”. The search was limited to articles published between 2009 and 2019. All original studies published in English, Portuguese or Spanish were eligible for this review. Incomplete articles, abstracts, theses, dissertations and studies of non-occupational risk factors associated with breast cancer were excluded from consideration.

After the literature search, the articles were read in order to identify the risk factors analyzed and the strength of their association with breast cancer. The information on the risk factors, authors, publication year, type of study, conclusions regarding significant or non-significant association and population were extracted from each study and compiled into tables. These findings were then discussed based on previous literature on the risk factors associated with breast cancer.

## RESULTS

The literature search retrieved 409 articles (11 from SciELO, 7 from LILACS and 391 from PubMed). After limiting the search to the aforementioned 10-year period, 369 articles were left. This was followed by abstract screening, application of selection criteria, and removal of duplicates, which resulted in the inclusion of 40 articles in the review.^3,4,6,12-46^ The risk factor, author, publication date, study type, conclusion and population analyzed in each study were entered into tables. Four occupational risk factors were identified in the studies: metals, chemical agents, radiation and circadian rhythm disruption.

The two articles that discussed an association between breast cancer and occupational exposure to metals identified cadmium as a possible carcinogen ^32,41^ (Table 1). One case-control study reported a significant association between cadmium and breast cancer, but noted that the influence of non-occupational risk factors was greater than that of occupational risk factors in the population studied.^41^

**Table 1:** Metal exposure as a risk factor for breast cancer

Risk factor	Author	Year	Type of study	Conclusion	Population
Cadmium	Rahim et al.^32^	2013	Meta-analysis	The prevalence of breast cancer may be an indicator of early genetic effects in populations exposed to cadmium.	Varied
	Peng et al.^41^	2015	Case-control study	Greater association between non-occupational exposure to cadmium and the progression of breast cancer.	Varied

Fourteen articles emphasized the association between breast cancer and exposure to chemical agents. These included agricultural pesticides, solvents, alkylphenols, motor fluid, petroleum, solder, ethylene oxide and aromatic hydrocarbons. The studies found that breast cancer was positively associated with exposure to pesticides, as well as most solvents and hydrocarbons (Table 2).

**Table 2:** Chemical exposures as risk factors for breast cancer

Risk factor	Author	Year	Type of study	Conclusion	Population
Pesticides	El-Zaemey et al.^42^	2013	Case-control study	Increased risk associated with exposure at a younger age.	Varied
	El-Zaemey et al.^43^	2014	Case-control study	No association between breast cancer and residential or occupational exposure to pesticides.	Varied
	Engel et al.^44^	2017	Cohort study	Organophosphate insecticides were associated with a risk of breast cancer. However, the association differed between wives and husbands.	Agricultural workers and their wives
	Khalis et al.^3^	2019	Case-control study	Occupational exposure may be associated with an increased risk of breast cancer.	Varied
Solvents	Peplonska et al.^45^	2010	Case-control study	Little evidence for an association between cancer and occupational exposure to organic solvents.	Varied
	Oddone et al.^6^	2014	Case-control study	Increased risk of breast cancer after exposure to chlorinated solvents.	Female electrical workers
	Ekenga et al.^26^	2014	Cohort study	Occupational exposure to pesticides prior to birth of the first child was related to an increased risk of breast cancer.	Varied
	Glass et al.^12^	2015	Case-control study	There may be an association between occupational exposure to low levels of aliphatic and aromatic solvents and a risk of breast cancer.	Varied
	Videnros et al.^4^	2019	Cohort study	Occupational exposure to chemical agents in general was associated with an increased risk of breast cancer. Exposure to organic solvents and diesel fuel were frequently observed.	Varied
Alkylphenols	Peremiquel-Trillas et al.^13^	2018	Case-control study	Modest association between cancer risk and occupational exposure to alkylphenolic coumpounds.	Varied
Motor fluid	Garcia et al.^14^	2018	Cohort study	Exposure to chemical lubricants with no oil content was associated with an elevated hazard ratio.	Female autoworkers
Oil and solder	Ekenga et al.^15^	2015	Cohort study	Organophosphate insecticides were associated with a risk of breast cancer. However, these associations differed between wives and husbands.	Varied
Ethylene oxide	Marsh et al.^16^	2019	Literature review	Epidemiological studies did not find this factor to be associated with an increase in breast cancer rates.	Varied
Aromatic hydrocarbons	Lee et al.^17^	2019	Case-control study	Increased risk of breast cancer, especially in women with a family history of this condition.	Varied

Nine studies discussed the role of radiation as a risk factor for the development of breast cancer (Table 3). The studies focused on two types of carcinogenic radiation: ionizing and non-ionizing radiation. Four articles emphasized the vulnerability of radiology technicians to the development of breast cancer. Two studies also discussed the risk of breast cancer in men exposed to radiation.

**Table 3:** Radiation as a risk factor for breast cancer

Risk factor	Author	Year	Type of study	Conclusion	Population
Ionizing radiation	Sigurdson et al.^19^	2010	Cohort study	Significant evidence of an association between breast cancer and exposure to ionizing radiation.	Radiology technicians
	Bhatti et al.^18^	2010	Case-control study	No clear association between radiation and an increase in breast cancer rates.	Radiology technicians
	Schonfeld et al.^20^	2010	Case-control study	Significant association between breast cancer and occupational exposure to radiation.	Radiology technicians
	Buitenhuis et al.^21^	2013	Case-control study	Low risk of breast cancer at the current level of occupational exposure to ionizing radiation.	Varied
	Preston et al.^22^	2016	Cohort study	Occupational radiation was positively associated with the risk of developing breast cancer.	Radiology technicians
Non-ionizing radiation	Chen et al.^23^	2013	Meta-analysis	Non-ionizing radiation may be related to an increased risk of breast cancer in women, especially those in the pre-menopausal period.	Varied
	Sun et al.^24^	2013	Meta-analysis	May be associated with an increased risk of male breast cancer.	Men
	Grundy et al.^25^	2016	Case-control study	Occupational exposure to magnetic fields for at least 30 years led to a near three-fold increase in breast cancer risk. However, the limitations of the study may have influenced these findings.	Men
	Chen et al.^26^	2010	Meta-analysis	No association.	Varied
	Li et al.^36^	2015	Cohort study	No association.	Textile workers

Twelve articles discussed circadian rhythm disruption and its association with breast cancer (Table 4). According to some of these studies, workers with rotating shifts, such as nurses, were likely to have an increased risk of developing breast cancer. Eight of the 12 articles demonstrated a positive association between alterations in the circadian rhythm and the development of breast cancer.

**Table 4:** Circadian rhythm disruption as a risk factor for breast cancer

Risk factor	Author	Year	Type of study	Conclusion	Population
Circadian rhythm disruption	Pronk et al.^28^	2010	Cohort study	Epidemiological evidence did not provide consistent support for this association.	Varied
	Lie et al.^29^	2011	Cohort study	Significantly higher risks were observed in nurses who worked for 6 consecutive night shifts over the course of 5 years.	Nurses
	Hansen et al.^30^	2012	Case-control study	Further evidence that night work can increase the risk of breast cancer.	Nurses
	Ijaz et al.^31^	2013	Meta-analysis	Insufficient evidence for a link between night work and breast cancer.	Varied
	He et al.^33^	2014	Meta-analysis	Circadian disruption is associated with an increased risk of breast cancer.	Varied
	Grundy et al.^34^	2014	Case-control study	Significant association between the two variables in the long term.	Varied
	Åkerstedt et al.^35^	2015	Cohort study	Increased risk of breast cancer in women, but only after relatively long exposure.	Varied
	Li et al.^36^	2015	Case-control study	Non-significant association.	Textile workers
	Cordina-Duverger et al.^37^	2017	Meta-analysis	Night work increases the risk of breast cancer in pre-menopausal women.	Varied
	Lee et al.^38^	2018	Review	Associations between breast cancer and night work were reported by several epidemiological investigations, including cohort studies, case-control studies and meta-analyses. However, the dose-response relationship was not clear.	Varied

Two articles focused specifically on one occupation: flight attendants (Table 5). These individuals are exposed to two different risk factors: circadian disruption due to jet lag and frequent exposure to cosmic radiation.

**Table 5:** Cosmic rays and circadian disruption as risk factors for breast cancer

Risk factor	Author	Year	Type of study	Conclusion	Population
Cosmic radiation	Liu et al.^39^	2016	Meta-analysis	Increased risk of breast cancer relative to the general population.	Flight attendants
Circadian disruption	Schubauer-Berigan et al.^40^	2015	Cohort study	Increased risk of cancer relative to the general population; however, the incidence of breast cancer was not related to cosmic radiation or circadian disruption.	Flight attendants

## DISCUSSION

Breast cancer is characterized by the presence of endocrine-responsive tumors with varying histopathological features, which can be influenced by environmental factors.^2^ They are significantly more common in women, although nearly 1% of men can also develop breast cancer.^47^

The association between breast cancer and occupational risk factors has drawn increasing attention from the scientific community. Recent advances in research methods have allowed for a more thorough analysis of specific exposures and their association with breast cancer. Metals, radiation, chemical substances and circadian disruption have all been identified as relevant risk factors in previous research.

## METALS

Some metals are toxic to humans. The carcinogenic effects of metals such as beryllium, arsenic, cadmium, mercury, nickel, lead and chromium have been demonstrated in previous research.^3,5^ The studies of metal exposure included in this review focused on cadmium, which has been classified as a carcinogen by the IARC.^48^ Its toxicity can affect estrogen and testosterone levels, which in turn can lead to breast cancer.^29,48,49^ Nevertheless, most of the studies reviewed found that exposure to cadmium was not significantly associated with the development of breast cancer.

## CHEMICAL COMPOUNDS

The association between chemical products and the etiology of breast cancer is a long-studied topic in the literature. Over 200 chemical products are known to have potentially carcinogenic effects on the mammary gland. These chemicals interact directly or indirectly with the endocrine system, mimicking or interfering with physiological processes. Chemical compounds that disrupt the endocrine system may affect breast growth, increase susceptibility to cancer or induce tumor growth by interfering with estrogen or progesterone regulation.

In this review, some of the substances identified as playing a potentially carcinogenic role in breast cancer were pesticides, solvents, petroleum products, hydrocarbons and ethylene oxide. Pesticides belong to a large group of chemical products that have prompted widespread concern and discussion in public health settings.^45^ In Brazil, they have been increasingly used for agricultural pest control and increase land productivity.^2^ However, the increasingly frequent and indiscriminate use of these substances can lead to serious health issues, especially for individuals working directly with these products, such as rural populations.^2,3^ Many pesticides have been found to mimic the effects of estrogen, which may increase the risk of breast cancer.^44^ The carcinogenic potential of these substances has been supported by in vitro and animal studies.^45^

Solvents are volatile organic compounds that can dissolve a wide variety of materials and are frequently used in occupational settings.^13^ Examples include Schauer benzene, other aromatic hydrocarbons and chlorinated solvents. Studies suggest that occupational exposure to solvents is associated with a 50% increase in breast cancer risk.^12^ These agents are genotoxic and may result in metabolic alterations as well as breast cancer.^12,13^

Alkylphenolic compounds are organic chemicals created during the production of alkylphenol ethoxylates (APEs), used especially as non-ionic surfactants, but with a wide range of other applications.^14^ Occupational exposure to chemical compounds can occur during the production of these substances, or as a result of exposure to domestic and industrial detergents, special dyes, pesticides, cosmetics and domestic cosmetics.^14^ Occupational exposure to alkylphenolic compounds was found to be significantly associated with breast cancer.

Polycyclic aromatic hydrocarbons are a large group of chemical compounds formed in the combustion of organic matter and are common environmental pollutants.^19^ However, prolonged and continuous exposure to these substances can increase the risk of breast cancer, especially in women with a family history of these malignancies.^19^

Direct exhaust gases and petroleum-derived products are complex mixtures of paraffinic, naphthenic and aromatic products refined from mineral oil.^15,16^ The carcinogenic properties of mineral oil, which is classified as carcinogenic to humans, are mainly attributed to its polycyclic hydrocarbon content. Diesel exhaust was classified by the IARC as carcinogenic to humans based on evidence of its association with an increased risk of lung cancer.^15^ However, we did not identify high-quality evidence on diesel and gasoline emissions and their association with breast cancer.

Ethylene oxide is a highly reactive chemical, used primarily as an intermediate in the synthesis of several industrial chemicals.^17^ Products derived from ethylene oxide include plastics, polyester fibers, detergents and ethylene glycol antifreeze.^17^ It is also used as an agricultural insecticide and in the sterilization of hospital equipment. The National Toxicology Program has classified ethylene oxide as a carcinogen based on epidemiological data.^17^ However, we found no evidence to support a correlation between breast cancer and exposure to ethylene oxide.

## RADIATION

Ionizing and non-ionizing radiation are natural phenomena. Both are considered carcinogenic, but the evidence of the association between breast cancer and ionizing radiation is far more extensive.^24,25^ The health effects of radiation are influenced by the duration and intensity of exposure. Ionizing radiation has been more thoroughly studied, and findings support its association with the risk for several types of malignancy, including breast cancer.^23^ Sources of radiation exposure include the particles and rays emitted by radioactive metals, high-tension equipment, nuclear reactions and cosmic phenomena. Examples of this type of radiation are gamma and X-rays, both of which are associated with malignancies such as breast cancer.^1,2^ Health care workers such as radiology technicians are especially vulnerable to this type of exposure. Non-ionizing radiation includes electromagnetic frequencies and microwaves, whose emission sources include household appliances.^25,47^ The duration of exposure appears to be a significant determinant of the effects of non-ionizing radiation. According to one of the studies reviewed, individuals exposed for at least 30 years had a nearly three-fold increase in the risk of breast cancer relative to individuals with a shorter duration of exposure.^47^

## CIRCADIAN RHYTHM DISRUPTION

Most epidemiological studies suggest a positive association between breast cancer and circadian disruption. Night work was classified as a Group 2A carcinogen by the IARC in 2007.^5^ Alterations in the circadian rhythm are thought to increase the risk of cancer by interfering with melatonin synthesis.^2,5^ A possible mechanism of action for this effect involves the suppression of nocturnal melatonin production due to exposure to artificial light, which can also influence the patterns of sex hormone production and, in turn, increase the risk of breast cancer.^2,5^ Two studies focused specifically on populations susceptible to circadian rhythm disruption due to rotating shift work.^33^ Another professional category that is vulnerable to circadian misalignment is that of flight attendants. These professionals often travel across time zones and experience the effects of jet lag.^50^ Flight attendants are also exposed to cosmic radiation. Long flights at high latitudes - that is, closer to the poles - are associated with a higher risk of exposure to cosmic rays.^50,51^

## CONCLUSIONS

The association between occupational exposures and the etiology of breast cancer has been examined by several studies which have supported the relationship between these variables and added to the growing interest in this issue demonstrated by scientists studying cancer and workers’ health issues.

Occupational exposure to chemicals, ionizing radiation and circadian disruption have proved to have the greatest association with breast cancer. Nevertheless, many studies have pointed to the difficulty in establishing a causal link between these factors due to the number of variables involved, such as the duration and intensity of exposure to risk factors.

There is a need for more studies centered on the relationship between breast cancer and occupational exposures. Such studies can provide a basis for the development of effective protection measures for these workers.
